# Ocean Heat Content Reveals Secrets of Fish Migrations

**DOI:** 10.1371/journal.pone.0141101

**Published:** 2015-10-20

**Authors:** Jiangang Luo, Jerald S. Ault, Lynn K. Shay, John P. Hoolihan, Eric D. Prince, Craig A. Brown, Jay R. Rooker

**Affiliations:** 1 Rosenstiel School of Marine and Atmospheric Science, University of Miami, Miami, Florida, United States of America; 2 Cooperative Institute for Marine and Atmospheric Studies, University of Miami, Miami, Florida, United States of America; 3 Southeast Fisheries Science Center, Sustainable Fisheries Division, Highly Migratory Species Branch, NOAA Fisheries, Miami, Florida, United States of America; 4 Department of Marine Biology, Texas A&M University, Galveston, Texas, United States of America; University of California Davis, UNITED STATES

## Abstract

For centuries, the mechanisms surrounding spatially complex animal migrations have intrigued scientists and the public. We present a new methodology using ocean heat content (OHC), a habitat metric that is normally a fundamental part of hurricane intensity forecasting, to estimate movements and migration of satellite-tagged marine fishes. Previous satellite-tagging research of fishes using archival depth, temperature and light data for geolocations have been too coarse to resolve detailed ocean habitat utilization. We combined tag data with OHC estimated from ocean circulation and transport models in an optimization framework that substantially improved geolocation accuracy over SST-based tracks. The OHC-based movement track provided the first quantitative evidence that many of the tagged highly migratory fishes displayed affinities for ocean fronts and eddies. The OHC method provides a new quantitative tool for studying dynamic use of ocean habitats, migration processes and responses to environmental changes by fishes, and further, improves ocean animal tracking and extends satellite-based animal tracking data for other potential physical, ecological, and fisheries applications.

## Introduction

Movements and seasonal migration ecology of vertebrate animals are largely driven by dynamic environmental features [1–4]. A strong relationship between oceanic temperature patterns and the migratory forays of many large pelagic tunas, billfishes, sharks, and tarpon has long been recognized [2, 5, 6]. Fronts and eddies are well known dynamic ocean habitats for fishes [7–9], but knowledge of their utilization has been based mostly on qualitative evidence from ecological theory or extractive fisheries data [7, 8, 10]. However, significant advances in the geolocation accuracy of electronic tags over the past two decades as a result of improved GPS and Argos positioning used on sea turtles[11, 12], sea birds[13, 14], and marine mammals[15, 16] have provided direct quantitative evidence that many marine vertebrates use ocean fronts and eddies as preferred habitats. In these studies modeled ocean current vectors and satellite remote sensing data (e.g., sea surface height (SSH, altimetry data); sea surface temperature (SST, infrared and microwave); and, sea surface chlorophyll (SSC, ocean color imagery) were used to distinguish these important ocean features. However, due to the relatively short surface intervals of most fishes, it has been difficult to fully utilize the advanced features of geolocation tags to produce accurate positions. A few recent studies have used these Argos geolocation tags on sharks [17, 18], striped marlin (*Kajikia audax*) [19] and Atlantic tarpon (*Megalops atlanticus*) [18], fishes which spend time at or near the surface. Nevertheless, due to their infrequent surfacing, geolocation data lack the temporal and spatial resolution required to study utilization of mesoscale fronts and eddies. As a default, some studies have used the light-level derived geolocations from electronic tags and ocean models to explore the large-scale ocean feature utilizations by marine predator fishes [20, 21].

**Fig 1:**
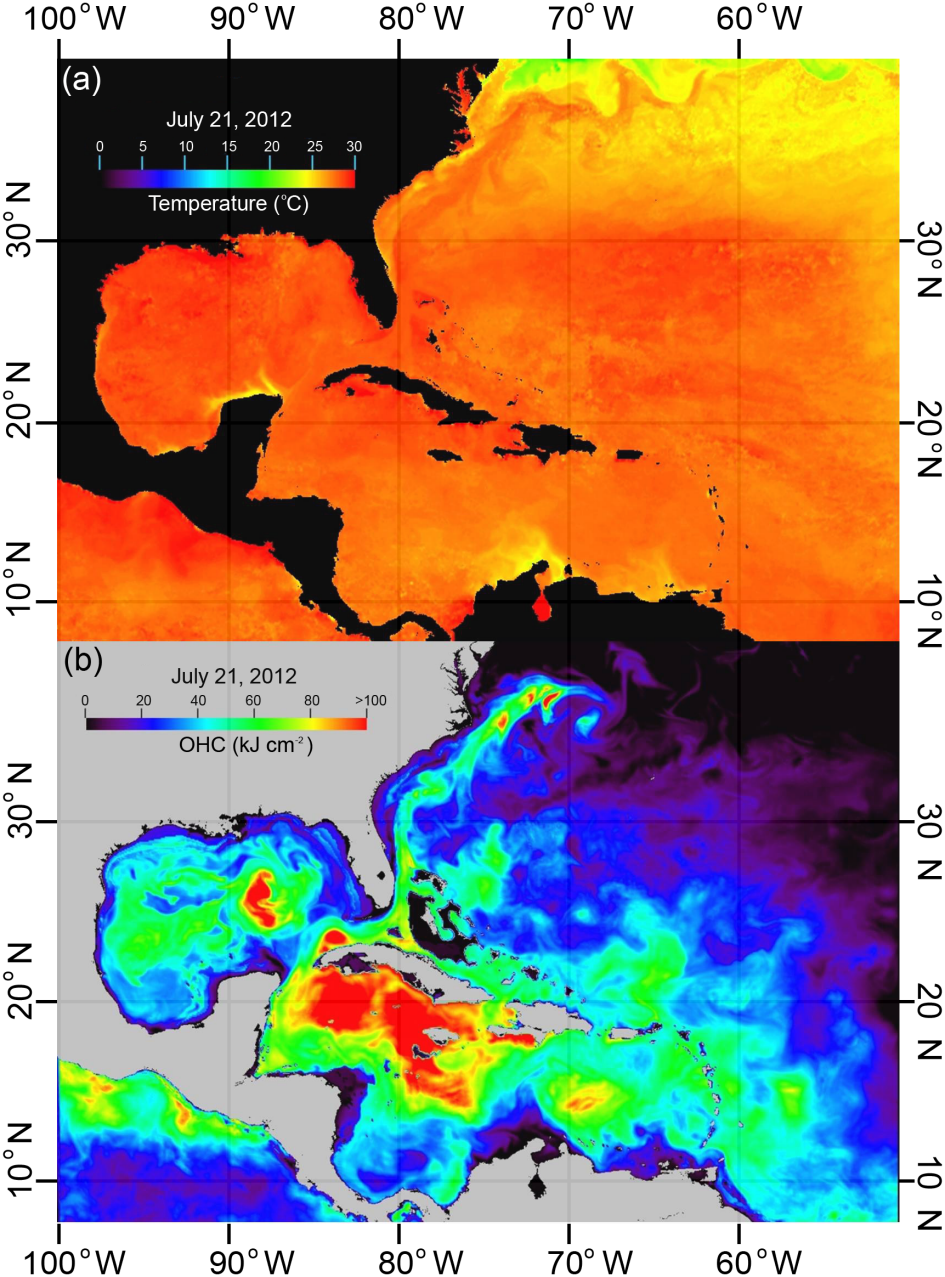
Ocean fronts and eddies revealed by ocean heat content (OHC). (a) Sea surface temperature (SST) and (b) OHC, on July 21, 2012.

Over the past decade, thousands of pop-up satellite archival tags (PSATs) with high frequency temperature‐depth-light sensors have been deployed on coastal and pelagic fishes [2, 6, 22, 23]. PSATs provide previously unavailable concurrent observations of fish movements and hydrographic conditions over relatively large ocean areas. Geolocation estimates between deployment and pop-up positions are derived from light, time and depth data recorded by the PSAT [24]. Inherent restrictions limit the accuracy of light-based longitudinal locations to ± 1 degree [24], and improvement of these estimates has been possible by re-processing the light-based positions using a Kalman filter (KF) and an unscented Kalman filter (UKF), then incorporating SST (KF-SST, UKF-SST) [25]. Despite advanced KF-SST and UKF-SST methods, the homogenous distribution of SST (Fig 1a) in tropical regions greatly denigrates usefulness of SST in refining movement tracks and in explicitly delineating fish utilization of dynamical ocean features. Among the variables (currents, SSH, SST, SSC) used to derive ocean features, only SST is directly recorded by the satellite tags. In this paper, we introduce a new method to estimate movements, and habitat association with ocean fronts and eddies, of electronically monitored marine animals, while avoiding the deficiencies inherent in SST-based geolocation estimates.

Ocean heat content (OHC), a fundamental measure of the heat stored in the upper surface layers, has been used for more than four decades by oceanographers and meteorologists to predict hurricane intensity [26, 27]. The minimum SST for hurricane formation is 26°C [27]. Expressed in kilo joules cm^-2^, OHC is estimated by integrating temperature from the depth of the 26°C isotherm to the ocean’s surface. OHC is typically obtained from satellite remote sensed SST data and vertical thermal structure measurements. For hurricane intensity forecasting, OHC is routinely estimated by an Atlantic regional temperature and salinity (SMARTS) climatology model that systematically merges daily thermodynamic fields from satellite remote sensing (i.e., radar altimetry and SST) with ocean observing system data [28, 29].

While evaluating the usefulness of OHC estimated from PSATs as a supplemental data source for SMARTS, we discovered that time-series OHC maps appeared to be superior for revealing pelagic fish habitats as compared to maps based solely on SST. During the tropical cyclone season (June 1 –November 1), SST maps of the Caribbean Sea and Gulf of Mexico regions of the central western Atlantic are generally featureless during most days (Fig 1a). In contrast, OHC maps revealed unseen structure in the upper thermocline of the water column due to the integrated heat content (Fig 1b). Thus, the goal of this research was to introduce and develop a new methodology using OHC to refine movement tracks of PSAT-monitored large pelagic fishes and to use examples to demonstrate how the refined movements tracks help us to explore the quantitative relationships between fish migration paths and dynamic oceanic features (fronts and eddies).

## Methods

Depth, temperature and light data were obtained from 137 PSATs deployed since 2004 on pelagic fishes for periods ranging from 10–180 d in the western central Atlantic Ocean from previously published [30–35] and unpublished studies. Light intensity data were processed using the global positioning software WC-AMP (Wildlife Computers, Redmond, WA, USA) to provide initial geolocations [36]. The light data provided sunrise and sunset times used to generate initial geolocation estimates. Then an SST-corrected Kalman filter [25, 37] (KF-SST or UKF-SST) was applied to refine light-derived geolocations. To relocate the points that were either on land or in shallow waters, we modified the estimates by also filtering depths based on 2 minute grid ETOP02 bathymetry data and the daily maximum depth from the tag [38].

**Fig 2:**
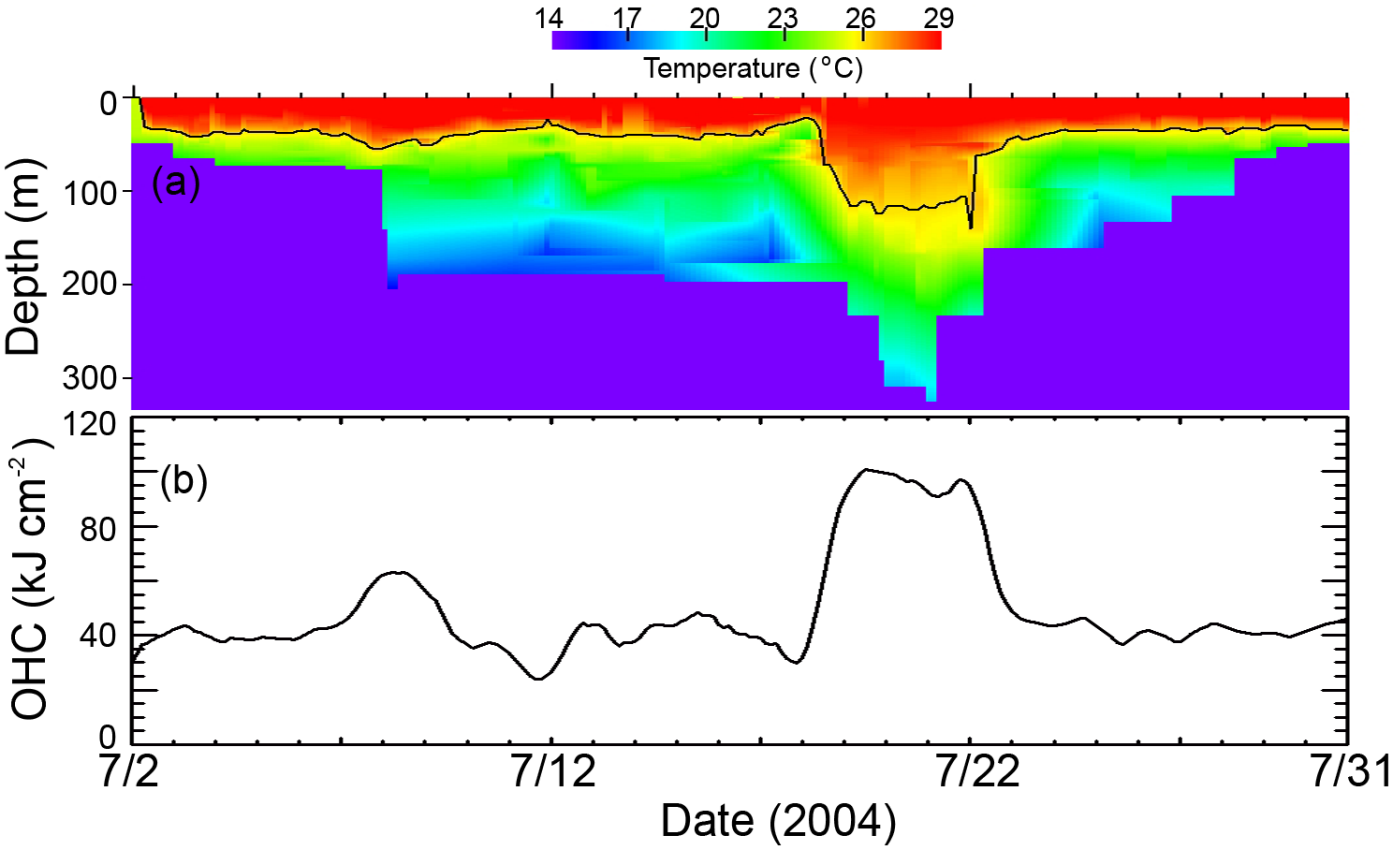
Estimates of OHC from satellite-tagged fish. (a) Profile of depth and temperature (PDT) from a PSAT tagged blue marlin in Gulf of Mexico during July 2004. The black line shows the depth of the 26°C isotherm. (b) OHC estimated from the above PDT profile.

Profiles of depth and temperature (PDT) along the migration tracks of individual fishes were mapped based on the continuous PSAT data binned at 3-h time intervals (Fig 2a). The midpoint of temperature minimum and maximum at each depth from PDT data was used to generate the vertical temperature profile of each tagged fish. The missing data bins were linearly interpolated with the nearest neighbor bins for both vertical and horizontal dimensions. However, in our analysis when a tag had, on average, more than three gaps of five days each, it was eliminated from further analysis. These eliminations comprised about 10% of the tags originally available to us. For fish track analysis, OHC values (Fig 2b) were calculated by integrating the thermal energy from the depths of the 26°C isotherm (D26) to the ocean surface according to the following equation [26]:

$$OHC=c_{p}\rho \int_{D26}^{0}(T_{z}-26{}^{\circ})dz$$ (1)

where, *c*_*p*_ is the specific heat constant of water, *ρ* is sea water density, and *T*_*z*_ is water temperature at depth *z*. For fishes inhabiting cooler waters, the OHC concept still holds, and simply requires adjusting the lower thermal limit downwards such as 20°C for tropical tunas and billfishes, or 10°C for temperate tunas (e.g., bluefin).

We generated OHC maps (*OHC*_*M*_) based on the global hybrid coordinate ocean model (HYCOM) by the US Navy Oceanographic Office (NAVO), using the real-time ocean data assimilation method [39]. We used the standard output, which has a spatial resolution of 0.08 deg (~7 km) and a daily time step. Global HYCOM has been shown to accurately resolve mesoscale processes such as meandering currents, fronts, and oceanic eddies [39]. We used the above equation and HYCOM temperature data to estimate the OHC, then we re-gridded the OHC values using linear interpolation methods to 2x2 min (0.033 x 0.033 degree) spatial resolution maps (*OHC*_*M*_) to match the ETOP02 [40] bathymetric grid.

**Fig 3:**
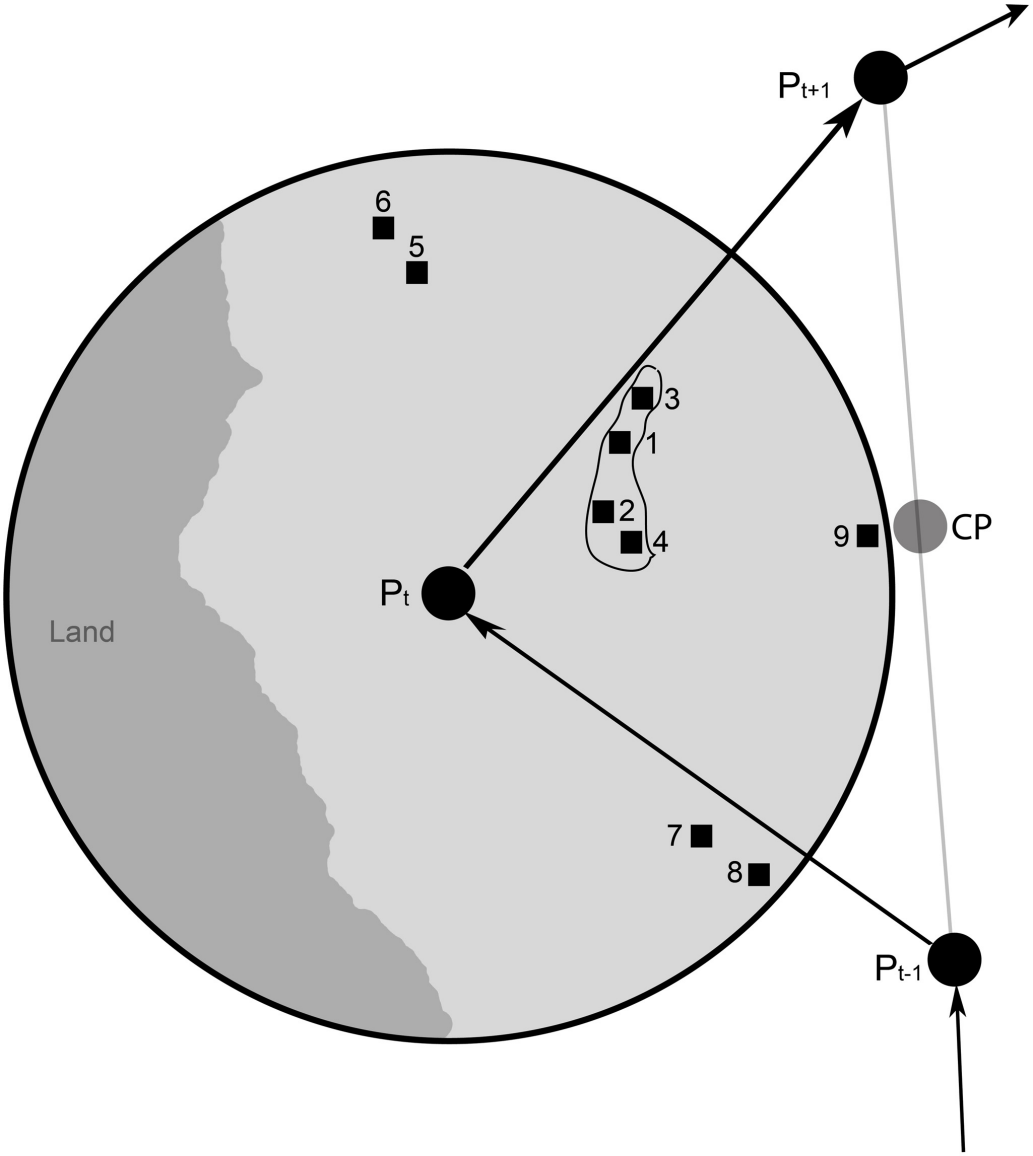
Search schematic of the genetic algorithm (GA). A circle of radius *R* is centered at the current position *P*_*t*_. The search area was constrained by land or shallow bathymetry. The numbers correspond to the nine scenarios outlined in the text.

We determined that migration tracks could be further optimized using OHC maps and a practical genetic optimization algorithm (GA) [41]. Our GA-OHC filtering method was similar to methods described previously [42]; however, we substituted SST maps with OHC maps. Here, we defined *OHC*_*T*_ as the OHC value estimated from the tag at time *t*, and *OHC*_*M*_ as the OHC value derived from the HYCOM model at position *P*_*t*_ at time *t* from either a KF-SST or UKF-SST derived track. In theory, if the position *P*_*t*_, *OHC*_*T*_ and *OHC*_*M*_ are estimated without error, then *OHC*_*T*_ and *OHC*_*M*_ should have the same value (i.e., Δ*OHC = OHC*_*M*_ *—OHC*_*T*_ *= 0*). If the position *P*_*t*_ is not accurate, Δ*OHC* will not equal to zero. Thus, we needed to find a new position $P_{t}^{\prime }$ where Δ*OHC* will be zero or minimized, a simple task if a single position was considered. However, an individual fish movement track over several months consists of hundreds to thousands of auto-correlated positions. Thus, the minimization procedure had to satisfy all track positions. To accomplish this goal, we applied a GA to minimize the sum of the square root of Δ*OHC* for all track positions. The optimization search method is schematically illustrated in Fig 3. At each time step, a search area is defined by a circle of a radius *R*, a variable ranging from 4–50 km that decreases as a function of generation time. *R* was also constrained by either land or shallow bathymetry (i.e., where water depths were less than the maximum depth of fish at the time *t*). The current position *P*_*t*_ was indicated by the dot at the center of the circle, and *P*_*t*-1_ was the fish’s position at previous time step, while *P*_*t*+1_ was the fish’s position at the subsequent time step. The mid-point between *P*_*t*-1_ and *P*_*t*+1_ is indicated by the center position (CP). In the simulations, the new position ($P_{t}^{\prime }$) takes a particular pixel according to one of nine scenarios:

1. The pixel with minimum absolute difference between the tag OHC (*OHC*_*T*_) and the model OHC (*OHC*_*M*_), that is, min[*OHC*_*T*_—*OHC*_*M*_].
2. The pixel with shortest distance to the center of the circle (*P*_*t*_) from the subset of minimum Δ*OHC* (a subset pixels where Δ*OHC* is > (Δ*OHC*_min_ + 0.1Δ*OHC*_min_).
3. The pixel with longest distance to the center of the circle (*P*_*t*_) from a subset of minimum Δ*OHC*.
4. The pixel with shortest distance to the CP of previous and next time step from a subset of minimum Δ*OHC*.
5. The pixel with the smallest OHC and shortest distance to the center of the circle *P*_*t*_.
6. The pixel with the smallest OHC and longest distance to the center of the circle *P*_*t*_.
7. The pixel with largest OHC and shortest distance to the center of the circle *P*_*t*_.
8. The pixel with largest OHC and longest distance to the center of the circle *P*_*t*_.
9. The pixel with shortest geometric average distance to previous (*P*_t-1_) and next (*P*_t+1_) position, that is, $\text{min}(\sqrt{D_{t-1}^{2}+D_{t+1}^{2}}\;)$.

The nine scenarios spread pixels over the entire range within the circle to overcome potential local minimizations during a given simulation. The basic idea was to use a GA to optimize the selection of the scenarios so as to not only minimize the sum square root of the error for an entire track, but also keep the distance between each position within the limit of known movement rates.

At the beginning of GA track simulation for a particular fish, 25 sequences (vectors) of movement rules were created for a movement track of *N* positions, where each vector will have a “gene code” of length *N* that is randomly generated, i.e., the movement rule for each time step was randomly selected from one of the 9 scenarios described above. At the end of each generation (i.e., completion of *N* movements), a selection function (SF) was calculated for each of the 25 tracks as the root-mean-square (*RMS*) error of OHC between the observed *OHC*_*T*_ and predicted *OHC*_*M*_ for all *N* positions as

$$RMS\;error=\sqrt{\frac{\sum_{1}^{N}{\left(OHC_{T}-OHC_{M}\right)}^{2}}{N-1}}$$ (2)

For the next generation, the three tracks (populations P1, P2, P3) with the lowest SFs were selected as parents and these retained the same genes (same movement rules) as their parents (P1, P2, P3). For the other 22 populations, the first 11 populations had the genes crossed (i.e., mixing movement rules) between P1 and P2 at a random crossing point, and then for the remaining 11 populations the genes were crossed between P1 and P3. Finally, for these 22 populations, 10% of their genes were allowed to mutate (i.e., change in movement rules) at random. Simulations ran until the SF reached a constant level within a specified tolerance (<0.01) [41, 42].

To increase speed and efficiency of the simulations, the *OHC*_*M*_ data were generated ahead of the tracking GA procedure and saved into a data base for all tags. Additional *OHC*_*M*_ data will be added to the database as they become available with time. In general, each simulation of a 100-day track took about 15 minutes to complete using currently available middle-tier specification desktop computers (i.e., Intel(R) Core™ i7 CPU @2.93 GHz).

To evaluate the accuracy of the GA-OHC method, a sinusoidal track extended over known *OHC*_*M*_ maps in the Gulf of Mexico (GoM) was generated as the “true” unbiased track, and the *OHC*_*M*_ values at the track positions were taken as the unbiased *OHC*_*T*_. Then, an altered track was generated by adding systematic sinusoidal biases of 0.5 degrees in latitude and random normal errors *N*(0,σ) on both the longitudes and latitudes of the “true” track. Finally, the GA-OHC method was applied to the altered track and unbiased *OHC*_*T*_ to generate an estimated track, then compared with the true track. The “true” track was 45 days long and was simulated bi-monthly using 2007 *OHC*_*M*_ data (Table 1) to evaluate the effects of different ocean conditions, i.e., seasonal changes in OHC. To evaluate the effect of OHC variation errors, an OHC average track was estimated by averaging all longitude and latitude values within a circle of a 0.5 degree radius at each point location where the *OHC*_*M*_ values were within *OHC*_*T*_ ±1 range. The mean and standard deviation of the differences between the average OHC track and the “true” track were calculated. Similarly, to repeat the validation simulations in a different geographic area, a 90 day blue marlin track from the South Atlantic Ocean was used as the “true” unbiased track, and the *OHC*_*M*_ values at those track positions were taken as the unbiased *OHC*_*T*_. Actual simulations were performed quarterly using 2005 *OHC*_*M*_ data.

**Table 1:** Validation results for GA-OHC filtered track estimates for Gulf of Mexico and South Atlantic Ocean. GoM validation simulations used bi-monthly OHC data on a 45-day sinusoidal track, and South Atlantic Ocean validation simulations used quarterly OHC data on 90-day blue marlin track. OHC variation (kJ cm^-2^) is the mean of the standard deviation (msd) of OHC values within a circle of 0.5 degree radius at each point location. OHC variation error presents the mean difference between the averaged OHC track and the “true” track, and the msd for each estimated location considering OHC errors of ±1, ±5 kJ cm^-2^.

OHC data period	OHC Variation msd	OHC error ±1				OHC error ±5			
		Longitude		Latitude		Longitude		Latitude	
		mean	msd	mean	msd	mean	msd	mean	msd
2007	Gulf of Mexico Track								
Jan-Feb	25.86	-0.007	0.146	-0.001	0.199	-0.009	0.155	-0.003	0.211
Mar-Apr	20.70	-0.012	0.186	-0.027	0.155	-0.011	0.197	-0.027	0.173
May-Jun	12.69	-0.025	0.180	0.014	0.186	-0.025	0.199	0.012	0.199
Jul-Aug	22.18	-0.008	0.211	-0.015	0.146	-0.006	0.221	-0.014	0.154
Sep-Oct	24.39	-0.016	0.145	-0.011	0.196	-0.018	0.156	-0.007	0.207
Nov-Dec	25.26	-0.017	0.166	-0.012	0.175	-0.013	0.175	-0.003	0.187
2005	South Atlantic Track								
Jan-Mar	7.86	-0.025	0.152	0.018	0.148	-0.019	0.182	0.024	0.171
Apr-Jun	8.57	-0.027	0.185	-0.008	0.144	-0.023	0.203	-0.006	0.162
Jul-Sep	7.57	0.008	0.179	-0.032	0.156	0.005	0.201	-0.033	0.182
Oct-Dec	6.84	0.002	0.173	-0.037	0.166	-0.002	0.197	-0.037	0.191

For the purposes of demonstration and application of the new method, the refined movements tracks of 124 pelagic fishes monitored with PSATs were used to explore the quantitative relationships between fish migration paths and oceanic features (fronts and eddies). We used the Belkin-O’Reilly algorithm [43] to compute the gradient of *OHC*_*M*_ and classified areas where gradients greater than 50 kJ cm^-2^ per grid cell were determined as zones of ocean discontinuities (ZOD). Fish track positions were then compared with the ZOD and all positions within one grid cell distance from ZOD were classified as being associated with ocean fronts and eddies. To simplify the presentation, the proportion of time that each fish spent associated with ZODs was calculated and categorized into three levels of eddy and front association: low (0–30%); medium (30–60%); and high (>60%). All numerical and visualization procedures were computed using IDL (Interactive Data Language, http://exelisvis.com), the procedures are available directly from the first author upon request. Fish handling and tagging procedures were followed Prince et al. [44], and was conducted under permits from the National Marine Fisheries Service Highly Migratory Species Division (TUNA-EFP-13-05, HMS-EFP-02-003), Florida Fish and Wildlife Conservation Commission (SAL-13-0062-SRP), National Park Service (EVER-2013-SCI-0064, DRTO-2014-SCI-0006), and the University of Miami Institutional Animal Care and Use Committee (Protocol # 12–221).

## Results

**Fig 4:**
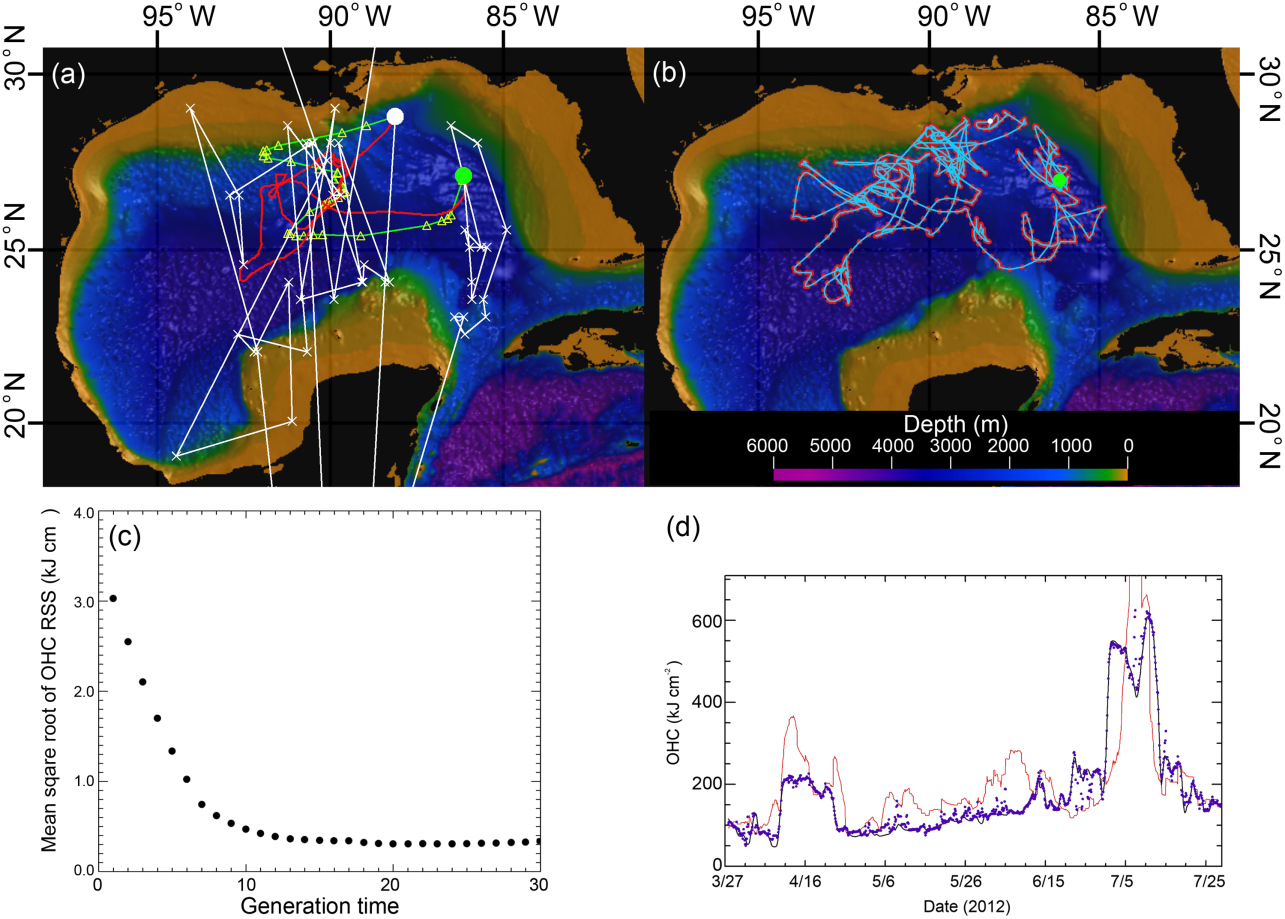
Movement tracks of a yellowfin tuna by different geolocation methods. (a) Geolocation estimates from light data (white x and line); light-based Kalman filtered track (yellow triangles and green line); UKF-SST track (red line). The white dot indicates the tag deployment location and the green dot indicates the tag pop-off location. (b) GA-OHC filtered track of the same fish. (c), The root-mean-square (*RMS*) error of OHC between the observed *OHC*_*T*_ and predicted *OHC*_*M*_ as a function of generation time. (d), Comparison of OHC values between UKF-SST and GA-OHC filtered tracks (black line is the *OHC*_*T*_ estimates from PSAT depth and temperature data; red line is *OHC*_*M*_ values at the location determined by the UKF-SST filtered track; and, purple dots are the *OHC*_*M*_ values at the location determined by the GA-OHC filtered track.

The UKF-SST filtered tracks (Fig 4a) were further refined with the GA that strives to minimize the RMS error between the observed *OHC*_*T*_ and predicted *OHC*_*M*_. The resultant GA-OHC filtered track (Fig 4b) revealed fish movements with much greater detail than those tracks produced by the UKF-SST method alone. The evolution of learning for the GA-OHC filter is shown by a decrease in the RMS as a function of generation time (Fig 4c). The strong similarity of the final OHC values is shown by comparing the *OHC*_*T*_ to the *OHC*_*M*_ (Fig 4d).

**Fig 5:**
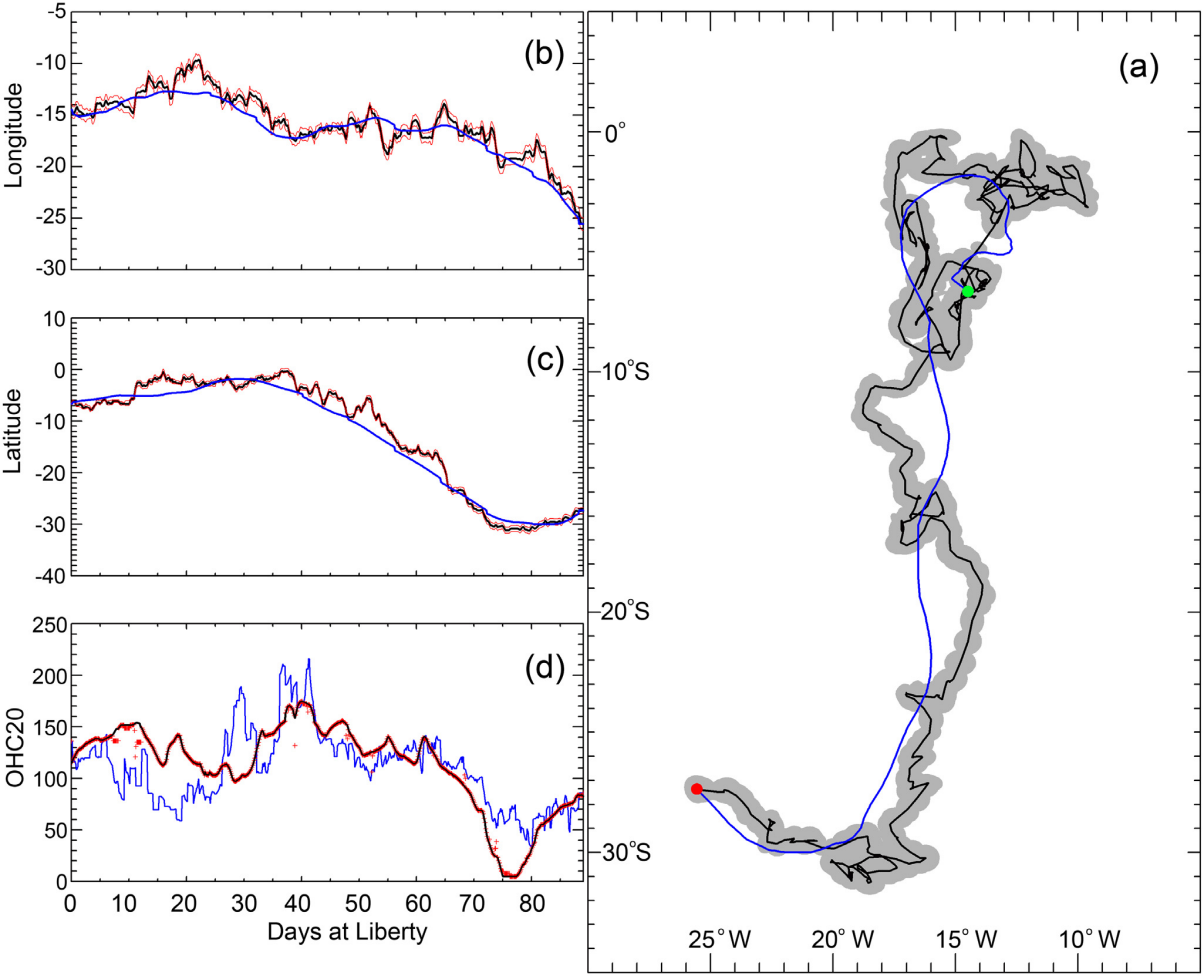
Movement tracks of a blue marlin in the South Atlantic. (a) UKF-SST track (blue line), GA-OHC filtered track (black line), and the 95% confidence intervals (grey shaded area). The green dot indicates the starting location (November 8, 2004) and red dot indicates the end location (Feb 5, 2005). (b) Longitude and (c) latitude before (blue) and after (black) GA-OHC filter as a function of days at liberty with 95% confidence intervals (red lines). (d) Comparison of OHC values between UKF-SST and GA-OHC filtered tracks. Black line shows the *OHC*_*T*_ estimates from PSAT depth and temperature data; blue line is *OHC*_*M*_ values at the locations determined by the UKF-SST filtered track; and, red crosses are the *OHC*_*M*_ values at the locations determined by the GA-OHC filtered track.

Another example is presented for a blue marlin track (Fig 5) in the South Atlantic Ocean. The GA-OHC filtered track (Fig 5a) revealed fish movements with much greater detail than those tracks produced by the UKF-SST method (blue line). The 95% confidence interval (grey shaded area) was calculated for each location using ±5 kJ cm^-2^ OHC variation around the estimated location. The confidence intervals are plotted as probability ellipses with 2 standard deviations of longitude, latitude as the major axes and angle of rotation determined by the covariance. The comparisons of longitude and latitude from the UKF-SST and GA-OHC track estimation methods are shown (Fig 5b and 5c). The final *OHC*_*M*_ values (red crosses) are optimized very close to the values of *OHC*_*T*_ (Fig 5d) as compared to the OHC values from the UKF-SST track (blue line).

**Fig 6:**
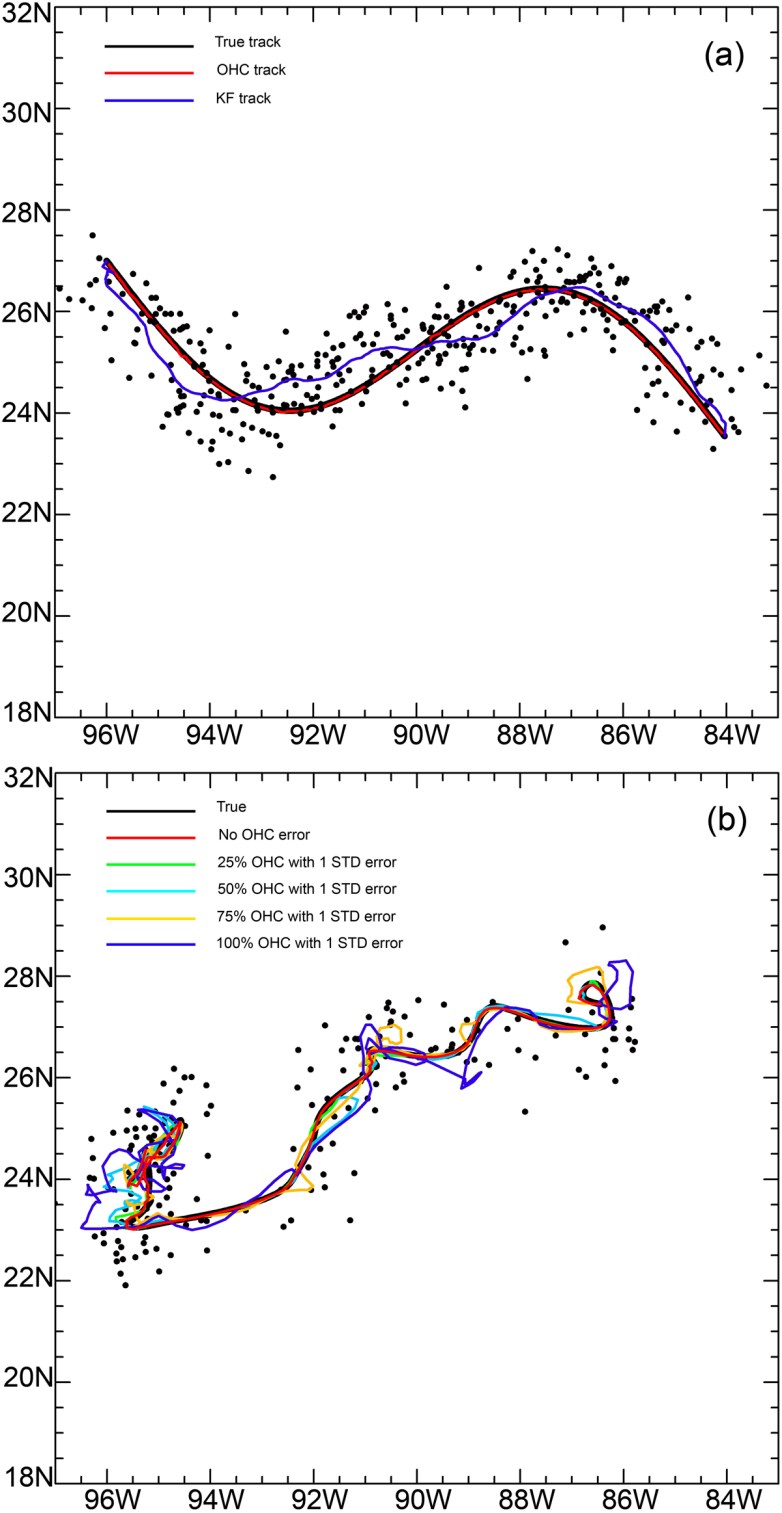
GA-OHC filter validation comparison. (a), Track based on a hypothetical sinusoidal curve (black), OHC track (red), and Kalman-filtered track (blue). (b), Track based on a tagged yellowfin tuna from April 7 to April 29, 2012 (black). GA-OHC filtered tracks were generated with ± 1 standard OHC error for 0% (red), 25% (green), 50% (light blue), 75% (yellow), 100% (blue) of the total positions. Dots are altered positions of “true” tracks.

The validation results of the simulated sinusoidal track (Fig 6a) showed that the GA-OHC filter improved the precision for determining the “true” track. The mean difference between the estimated track and the “true” track was less than 0.001 degrees and less than 0.01 degrees standard deviation for longitude and latitude during all periods of the year. The results were confirmed through validation of tagged fish that showed similar results in accuracy (Fig 6b). The effect of OHC variation (Table 1) was demonstrated by comparing the OHC average track to the “true” track. The mean differences in both longitude and latitude for all validation simulations were less than 0.04 degree, which is consistent with the 2-minute grid resolution (0.033 degree) of the model. For the GoM track and all periods of the year, the mean of the standard deviation (msd) ranged from 0.145 to 0.211, and 0.154 to 0.221, with OHC variation of ±1 and ±5 kJ cm^-2^, respectively. For the South Atlantic Ocean track, the msd ranged from 0.144 to 0.185, and 0.162 to 0.203, with OHC variation of ±1 and ±5 kJ cm^-2^, respectively. The OHC msd values (Table 1) within the circle of the 0.5 degree radius from each GA-OHC position ranged from 12.74 to 25.81 kJ cm^-2^, and 6.84 to 8.57 kJ cm^-2^, for the GoM and South Atlantic Ocean, respectively. This suggests the presence of a year-round OHC gradient for both geographic areas. On average, the OHC gradient is much steeper for the GoM than the South Atlantic Ocean.

**Fig 7:**
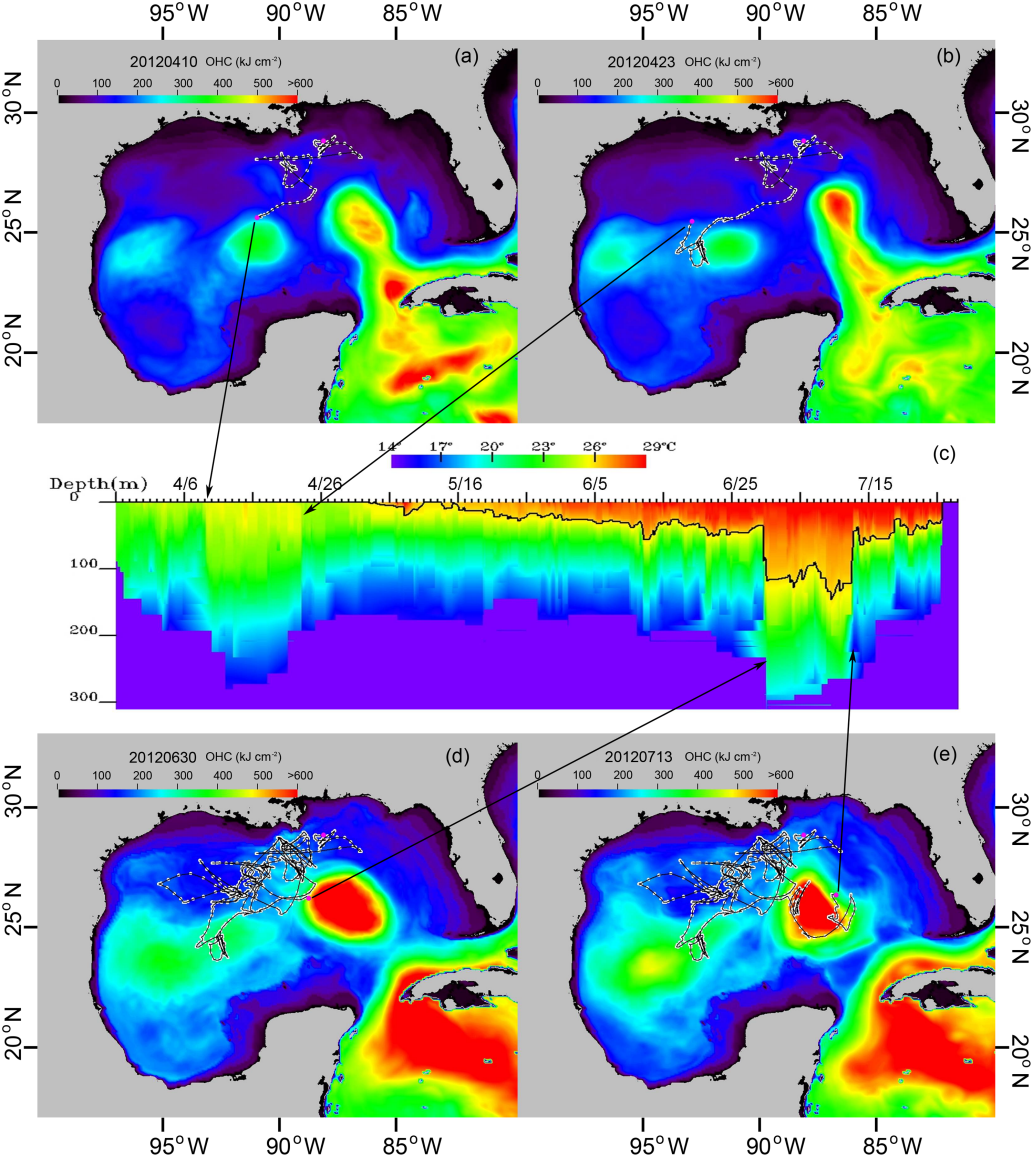
OHC refined movement track of a yellowfin tuna in the Gulf of Mexico. (a) Track position overlain on the OHC map for April 10, 2012. (b) Track position overlain on the OHC map for April 23. (c) PDT from the tagged yellowfin tuna. (d) Track positions overlain on OHC map for June 30. (**e**) Track positions overlain on OHC map for July 13.

The GA-OHC filtered tracks revealed detailed movements of fishes that were not apparent from track estimates using UKF-SST alone. For example, the detailed movements of one yellowfin tuna (*Thunnus albacares*) around an eddy and the Loop Current (LC) in the GoM are shown (Fig 7). From March 27 to April 9, 2012, this tuna was moving among weak fronts off the Mississippi River (Fig 7a) before reaching an eddy centered in the GoM on April 10^th^. The eddy structure is discernible on the *OHC*_*M*_ and PSAT temperature-depth profile (Fig 7c). The fish left the eddy on April 23 (Fig 7b). For the next two months it moved between weak fronts on the continental slope. On June 30^th^, it arrived on the western side of a newly formed eddy that was shed from the LC (Fig 7d), then proceeded around the boundary to the east side of the eddy by July 13 (Fig 7e).

**Fig 8:**
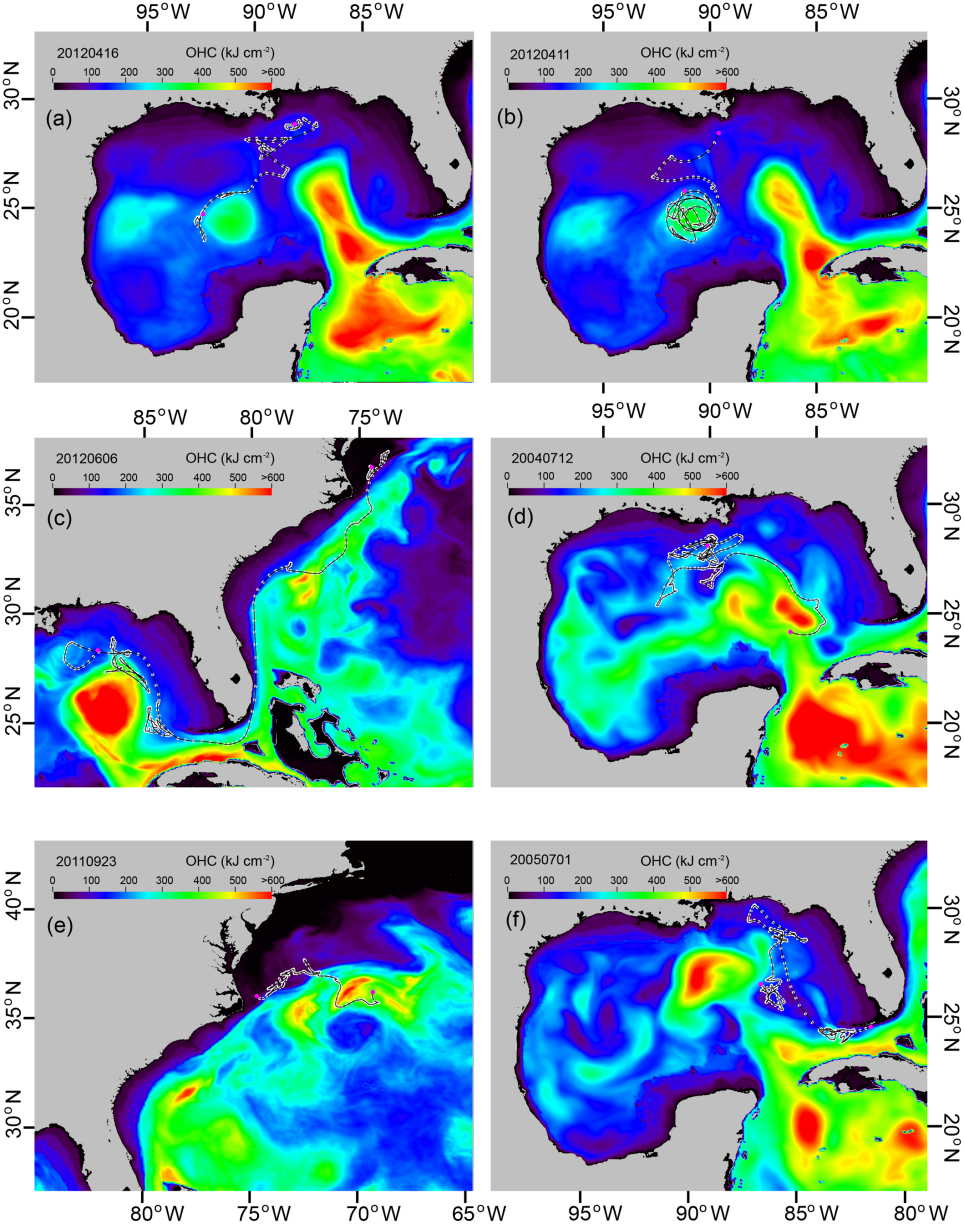
Front and eddy utilization by pelagic tunas and billfishes. (a) A yellowfin tuna along the edge of an eddy in the center of GoM on April 14, 2012. (b) Another yellowfin tuna along the edge of an eddy in the center of GoM on March 23, 2012. (c) A bluefin tuna along the eastern edge of the Loop Current (LC) and later along the western edge of the Gulf Stream from April 26 to June 6, 2012. (d) A blue marlin along the eastern edge of the LC on July 12, 2004. (e) A white marlin along the edge of the Gulf Stream on September 23, 2011. (f) A sailfish at the edge of Florida Current and the eastern edge of the LC from May 10 to July 1, 2005.

The 124 pelagic fishes monitored with PSATs, including yellowfin tuna, blue marlin (*Makaira nigricans*), white marlin (*Tetrapturus albidus*), and sailfish (*Istiophorus platypterus*), exhibited a strong preference for fronts and eddies, the dominant features of ocean discontinuities (Table 2). From 2009 to 2013 the area of “ocean discontinuities” accounted for about 18% of total available habitat (range of 10.6% to 27.1%, mean of 17.9%, standard deviation of 3.8%) in the GoM and Atlantic Ocean (50°W to 100°W longitude, 0° to 50°N latitude). More than 50% of these tagged fish spent greater than 60% of their time associated with ocean discontinuities. To further highlight these associations, we provide here some individual illustrative tracks. The yellowfin tuna previously described showed clear preferences for the edges of the eddy (Fig 8a) and the LC (Fig 7e). Another yellowfin tuna exhibited a high affinity for the edge of the same eddy located in the center of the GoM (Fig 8b). Remarkably, this particular tuna swam around the periphery of the eddy many times over 20 days (March 22 to April 11), rarely crossing over the eddy. One bluefin tuna was observed using the eastern edge of the LC while in the GoM, then the western edge of the Gulf Stream as it migrated north in the western North Atlantic Ocean (Fig 8c). Front and eddy edge use were also observed for billfishes. Blue marlin appeared to prefer the edges of cold- and warm-core eddies, and also the LC (Fig 8d). Similarly, the edge of the Gulf Stream front was used by white marlin off Cape Hatteras (Fig 8e). Finally, a sailfish monitored in the Florida Keys used the edge of the Florida Current, then transitioned to the edge of the LC while moving north-northwest in the GoM (Fig 8f).

**Table 2:** Front and eddy utilization by satellite-tracked fish. Summary of proportion of total time at liberty associated with fronts and eddies by individual PSAT-tagged yellowfin tuna, blue marlin, white marlin, and sailfish based on GA-OHC filtered tracks. Individual tracks were grouped into high, moderate and low utilization.

Species	Number of fish	Days at Liberty	High >60%	Moderate 30–60%	Low <30%
Yellowfin tuna [34]	59	3,287	41	16	2
Blue marlin [30, 45]	29	2,730	24	5	0
White marlin [35]	28	3,117	16	12	0
Sailfish [46]	8	988	8	0	0
**Species Combined**	**124**	**10,122**	**65**	**28**	**2**
**Percent**			**52.4%**	**22.6%**	**1.6%**

## Discussion

Our validation results (Fig 6 and Table 1) clearly demonstrated that the GA-OHC is effective in elucidating the “true” track from biased location data. The GA technique was very efficient in addressing the complexities that have hindered calculus-based methods [41]. No previous fish tracking studies [47, 48] have incorporated GA methods to match tag SST with satellite SST data for the purpose of improving location estimates from the light level data. Teo et al. [47] reported a mean error ranging from 0.73 to 1.41 degrees and standard deviation ranging from 0.54 to 1.28 degrees, while no error estimates were provided by Domeier et al. [48]. Nielsen et al. [37] and Lam et al. [25] included SST data into the KF and UKF state-space model to estimate the most probable tracks. They reported a standard deviation of 0.5 degree in longitude, and 1.2 degree in latitude. Our validation results are not directly comparable to earlier studies as regards accuracy basically because we lacked Argos positions from double-tagged fish for direct comparisons; however, these results did give a relative sense of the method’s precision. Our GA-OHC method had mean differences less than 0.04 degrees between the “true” and estimated tracks for both longitude and latitude, and msd of 0.15–0.22 degrees (~15–25 km) for both longitude and latitude when considering OHC errors of ±1 and ±5 kJ cm^-2^ (Table 1). This represents a large reduction in position error, which is much smaller than mesoscale eddies (<100 km [49, 50]). While all of these methods do not necessarily require the presence of a spatial gradient in the data *per se* (i.e., SST or OHC), it greatly facilitates model fitting. In tropical and sub-tropical ocean regions SST data are more or less uniform throughout large areas (Fig 1a), while the OHC data revealed the ocean with mesoscale features such as fronts and eddies (Fig 1b). Our study showed that OHC gradients are present year-round in the GoM and South Atlantic Ocean (Table 1) with much higher OHC gradients in the GoM. When the objective is to explore the relationship between fish migration paths and ocean features, an exact geolocation (i.e., errors in SST or OHC maps) may be less important than when the fish are in the proximity of these features. Use of an OHC gradient of 50 kJ cm^-2^ to discriminate ocean features should produce robust estimates of fish associations with those dynamic ocean features.

Besides SST and OHC, other variables such as SSH, SSC and ocean current vectors, could be used to differentiate mesoscale ocean features in oceanographic and ecological studies [10, 13, 43, 51]. However, we did not use them for fish tracking purposes because these variables were not recorded or estimated by any satellite fish tags under present technologies. The OHC is a unique metric because it can be estimated from ocean circulation models and satellite tags, despite not being measured or recorded directly by the sensors presently aboard either satellites or the electronic fish tags. By combining the power of the genetic algorithm and OHC, the GA-OHC filtering method could provide, with a little refinement, a powerful tool for improving the accuracy of movement tracks for satellite tagged fishes.

Through the novel application of OHC in this study, a clear connection emerged between the behaviors of fish and spatiotemporal dynamics of oceanographic features. The GA-OHC filtered tracks suggested that fishes use oceanographic habitat features as ecological road maps to guide migrations. It was not clear whether they were responding directly to the physical features, or were searching for prey, where the prey also may have responded to the physical features, or both. Ocean fronts and eddies support high biological productivity at all trophic levels [7, 52], and are well known as “hotspots” of marine fish habitats [4, 53]; however, most of this knowledge has been garnered from ichthyoplankton surveys [54], recreational and commercial fisheries catch data, or ecological theory [7, 8]. Compared to UKF-SST tracks (Fig 4a), the GA-OHC track methodology (Fig 4b) greatly improved the level of detail elucidating fish movements in a dynamic ocean environment (Figs 7 and 8) over those previously presented for large pelagic fishes monitored with electronic tags [2]. We believe the reason GA-OHC generated more realistic movement tracks and revealed previously unknown levels of front and eddy use during fish migrations was not only due to higher track resolution, but also because OHC integrates the vertical thermal properties of oceanographic features in a way that is similar to what fishes do when they naturally swim through those features and use ambient information to discriminate zones of ocean discontinuity.

These detailed movement tracks present tremendous implications for the use of satellite tagging data to study fish behavior in relation to habitat features in the seemingly monotonous seascape of the vast ocean. For example, the GoM is the only known spawning ground for the bluefin tuna in the western North Atlantic Ocean. The bluefin tuna track (Fig 8c) showed the fish was located at the eastern edge of the LC, an area known for spawning activity based on high concentrations of newly hatched larvae [54] and adult bluefin tuna catches [55]. The idea that ocean fronts and eddies are “hotspots” of marine fish habitats is not necessarily a new concept [4, 9, 53] *per se*, what is novel is the explicit empirical association of fishes with these prominent oceanographic features. The inherent physical characteristics of fronts and eddies are known to concentrate, retain, and enhance the productivity of living resources [7, 8, 56]. While fronts and eddies may only account for a small fraction of the vast ocean, they play a major role in shaping the seascape of marine ecosystems as key zones of intense predator-prey interactions across every trophic level.

This study demonstrates that *OHC*_*T*_ estimated from PSAT data are precise and can be useful as another integrated data source to refine OHC maps and improve the prediction of oceanic states in models. Development of a satellite tag capable of GPS quality location and instantaneous transmissions of position and multi-sensor data could be highly beneficial to the SMARTS system, resolving smaller-scale oceanic variability in the OHC maps that could improve hurricane intensity forecasting [28]. Improving OHC measurements at the boundaries of fronts and eddies is essential to the generation of accurate and precise OHC maps. Because large pelagic fishes have an affinity for these areas, using a network of satellite-tagged fish to monitor OHC values and augment other platforms is highly advantageous and cost-effective, as compared to the more traditional use of XBTs, floats, drifters, and gliders that are very difficult to keep in these boundary zones. Fish could greatly assist the resolution of structures around oceanic fronts and eddies at much smaller scales where important air-sea interactions occur under strong winds.

Given the potential availability of this new GA-OHC method, re-analyses of existing satellite tagging data will almost certainly shed new light on movement and behavior, and afford scientists a powerful tool to develop and investigate new hypotheses. Besides facilitating improvements in track prediction, OHC maps may also be applicable to other aspects of marine ecosystem research. These might include, for example, replacement of SST maps with OHC as a primary ecosystem indicator variable to help define, identify, and model essential habitats and improve estimates of the spatial abundance distribution for fishery abundance forecasts [57], conservation and management policy [58, 59], reproductive ecology [60], and climate changes [61].

## Supporting Information

S1 Movie Animated migration track of yellowfin tuna. Filename: ftp://ftp.rsmas.miami.edu/users/jluo/OHC/track/YFT_OHC_track.mp4.
(MP4)

S2 Movie Animated migration track of bluefin tuna. Filename: ftp://ftp.rsmas.miami.edu/users/jluo/OHC/track/BFT_OHC_track.mp4.
(MP4)

## References

[1] Leggett WC. The ecology of fish migrations. Annu Rev Ecol Syst, (no 8), 285–308, (1977). 1977.; 06294560.

[2] Block BA, Jonsen ID, Jorgensen SJ, Winship AJ, Shaffer SA, Bograd SJ, et al Tracking apex marine predator movements in a dynamic ocean. Nature. 2011;475(7354):86–90. 10.1038/nature10082 21697831

[3] Rooker JR, Secor DH, De Metrio G, Schloesser R, Block BA, Neilson JD. Natal homing and connectivity in Atlantic bluefin tuna populations. Science. 2008;322(5902):742–4. 10.1126/science.1161473 18832611

[4] Sydeman WJ, Brodeur RD, Grimes CB, Bychkov AS, McKinnell S. Marine habitat “hotspots” and their use by migratory species and top predators in the North Pacific Ocean: Introduction. Deep-Sea Res Pt II. 2006;53(3–4):247–9. 10.1016/j.dsr2.2006.03.001

[5] Luo J, Ault JS, Larkin MF, Barbieri LR. Salinity measurements from pop-up archival transmitting (PAT) tags and their application to geolocation estimation for Atlantic tarpon. Mar Ecol Prog Ser. 2008;357:101–9.

[6] Luo J, Ault JS. Vertical movement rates and habitat use of Atlantic tarpon. Mar Ecol Prog Ser. 2012;467:167–80.

[7] Olson DB. Biophysical dynamics of ocean fronts In: Robinson AR, McCarthy JJ, Rothschild BJ, editors. The Sea. New York: John Wiley & Sons; 2002 p. 187–218.

[8] Owen RW. Fronts and eddies in the sea: Mechanisms, interactions and biological effects In: Longhurst AR, editor. Analysis of Marine Ecosystems. London: Academic Press; 1981 p. 197–233.

[9] Belkin IM, Hunt GL Jr, Hazen EL, Zamon JE, Schick RS, Prieto R, et al Fronts, fish, and predators. Deep-Sea Research Part II: Topical Studies in Oceanography. 2014;107:1–2.

[10] Zainuddin M, Saitoh K, Saitoh S-I. Albacore (Thunnus alalunga) fishing ground in relation to oceanographic conditions in the western North Pacific Ocean using remotely sensed satellite data. Fish Oceanogr. 2008;17(2):61–73. 10.1111/j.1365-2419.2008.00461.x

[11] Mansfield KL, Wyneken J, Porter WP, Luo J. First satellite tracks of neonate sea turtles redefine the 'lost years' oceanic niche. Proc R Soc B Biol Sci. 2014;281(1781). 10.1098/rspb.2013.3039 PMC3953841 24598420

[12] Shillinger GL, Di Lorenzo E, Luo H, Bograd SJ, Hazen EL, Bailey H, et al On the dispersal of leatherback turtle hatchlings from Mesoamerican nesting beaches. Proc R Soc B Biol Sci. 2012;279(1737):2391–5. 10.1098/rspb.2011.2348; 20367999. PMC3350667 22378803

[13] Cotté C, Park Y-H, Guinet C, Bost C-A. Movements of foraging king penguins through marine mesoscale eddies. Proc R Soc B Biol Sci. 2007;274(1624):2385–91.; 17669726. 10.1098/rspb.2007.0775 PMC2274980 17669726

[14] Sabarros PS, Grémillet D, Demarcq H, Moseley C, Pichegru L, Mullers RHE, et al Fine-scale recognition and use of mesoscale fronts by foraging Cape gannets in the Benguela upwelling region. Deep-Sea Research Part II: Topical Studies in Oceanography. 2014;107:77–84.

[15] Bestley S, Jonsen ID, Hindell MA, Guinet C, Charrassin J-B. Integrative modelling of animal movement: incorporating in situ habitat and behavioural information for a migratory marine predator. Proc R Soc B Biol Sci. 2013;280(1750):20122262 10.1098/rspb.2012.2262; 20368334. PMC3574443 23135676

[16] Bailleul F, Cotte C, Guinet C. Mesoscale eddies as foraging area of a deep-diving predator, the southern elephant seal. Mar Ecol Prog Ser. 2010;408:251–64. 10.3354/meps08560; 14409270.

[17] Hammerschlag N, Gallagher AJ, Wester J, Luo J, Ault JS. Don't bite the hand that feeds: assessing ecological impacts of provisioning ecotourism on an apex marine predator. Funct Ecol. 2012;26:567–76.

[18] Hammerschlag N, Luo J, Irschick DJ, Ault JS. A comparison of spatial and movement patterns between sympatric predators: bull sharks (Carcharhinus leucas) and Atlantic tarpon (Megalops atlanticus). PloS ONE. 2012;7(9):1 10.1371/journal.pone.0045958; 23049904. PMC3458817 23049904

[19] Holdsworth JC, Sippel TJ, Block BA. Near real time satellite tracking of striped marlin (*Kajikia audax*) movements in the Pacific Ocean. Mar Biol. 2009;156(3):505–14.

[20] Teo SLH, Boustany AM, Block BA. Oceanographic preferences of Atlantic bluefin tuna, *Thunnus thynnus*, on their Gulf of Mexico breeding grounds. Mar Biol. 2007;152:1105–19.

[21] Sims DW, Witt MJ, Richardson AJ, Southall EJ, Metcalfe JD. Encounter success of free-ranging marine predator movements across a dynamic prey landscape. Proc R Soc B Biol Sci. 2006;273(1591):1195–201.; 16720391. 10.1098/rspb.2005.3444 PMC1560279 16720391

[22] Lutcavage ME, Brill RW, Skomal GB, Chase BC, Howey PW. Results of pop-up satellite tagging of spawning size class fish in the Gulf of Maine: do North Atlantic bluefin tuna spawn in the mid-Atlantic? Can J Fish Aquat Sci. 1999;56(2):173–7.

[23] Hussey NE, Kessel ST, Aarestrup K, Cooke SJ, Cowley PD, Fisk AT, et al Aquatic animal telemetry: A panoramic window into the underwater world. Science. 2015;348(6240):10. 10.1126/science.1255642 26068859

[24] Hill RD, Braun MJ, editors. Geolocation by light level—the next step: latitude Proceedings of the Symposium on Tagging and Tracking Marine Fish with Electronic Devices, February 7–11, 2000, East-West Center, University of Hawaii; 2001: Kluwer Academic, Dordrecht 468 pp.

[25] Lam CH, Nielsen A, Sibert JR. Improving light and temperature based geolocation by unscented Kalman filtering. Fish Res. 2008;91:15–25.

[26] Leipper DF, Volgenau D. Hurricane heat potential of the Gulf of Mexico. J Phys Oceanogr. 1972;2:218–24.

[27] Palmen E. On the formation and structure of tropical cyclones. Geophysika. 1948;3:26–38.

[28] Shay LK, Brewster JK. Oceanic Heat Content Variability in the Eastern Pacific Ocean for Hurricane Intensity Forecasting. Monthly Weather Review. 2010;138(6):2110–31. 10.1175/2010MWR3189.1; 13209998.

[29] Meyers PC, Shay LK, Brewster JK. Development and analysis of the systematically merged Atlantic regional temperature and salinity climatology for oceanic heat content estimates. Journal of Atmospheric and Oceanic Technology. 2014;31(1):131–49. 10.1175/JTECH-D-13-00100.1; 19024038.

[30] Goodyear C, Luo J, Prince ED, Hoolihan JP, Snodgrass D, Orbesen ES, et al Vertical habitat use of Atlantic blue marlin Makaira nigricans: interaction with pelagic longline gear. Mar Ecol Prog Ser. 2008;365:233–45.; 8416925.

[31] Kraus RT, Wells RJD, Rooker JR. Horizontal movements of Atlantic blue marlin (*Makaira nigricans*) in the Gulf of Mexico. Mar Biol. 2011;158:699–713. 10.1007/s00227-010-1593-3

[32] Hoolihan JP, Luo J, Goodyear CP, Orbesen ES, Prince ED. Vertical habitat use of sailfish (Istiophorus platypterus) in the Atlantic and eastern Pacific, derived from popaup satellite archival tag data. Fish Oceanogr. 2011;20(3):192–205. 10.1111/j.1365-2419.2011.00577.x; 14666493.

[33] Hoolihan JP, Luo J, Abascal FJ, Campana SE, De Metrio G, Dewar H, et al Evaluating post-release behaviour modification in large pelagic fish deployed with pop-up satellite archival tags. Ices Journal of Marine Science. 2011;68(5):880–9. 10.1093/icesjms/fsr024

[34] Hoolihan JP, Wells RJD, Luo J, Falterman B, Prince ED, Rooker JR. Vertical and Horizontal Movements of Yellowfin Tuna in the Gulf of Mexico. American Fisheries Society; 2014 p. 211–22.

[35] Hoolihan JP, Luo J, Snodgrass D, Orbesen ES, Barse AM, Prince ED. Vertical and horizontal habitat use by white marlin Kajikia albida (Poey, 1860) in the western North Atlantic Ocean ICES Journal of Marine Science. 2015; 10.1093/icesjms/fsv082

[36] Sibert JR, Musyl MK, Brill RW. Horizontal movements of bigeye tuna (*Thunnus obesus*) near Hawaii determined by Kalman filter analysis of archival tagging data. Fish Oceanogr. 2003;12(3):141–51.

[37] Nielsen A, Bigelow KA, Musyl MK, Sibert JR. Improving light-based geolocation by including sea surface temperature. Fish Oceanogr. 2006;15:314–25.

[38] Hoolihan JP, Luo J. Determining summer residence status and vertical habitat use of sailfish (*Istiophorus platypterus*) in the Arabian Gulf. ICES J Mar Sci. 2007;64:1791–9.

[39] Chassignet EP, Hurlburt HE, Smedstad OM, Halliwell GR, Hogan PJ, Wallcraft AJ, et al The HYCOM (HYbrid Coordinate Ocean Model) data assimilative system. J Mar Systems. 2007;65(1–4 SPEC. ISS.):60–83.

[40] Anon. U.S. Department of Commerce, National Oceanic and Atmospheric Administration, National Geophysical Data Center. *2-minute Gridded Global Relief Data (ETOPO2v2)* http://www.ngdc.noaa.gov/mgg/fliers/06mgg01.html/. 2006.

[41] Haupt RL, Haupt SE. Practical Genetic Algorithms. Second ed Hoboken, New Jersey: John Wiley & Sons, Inc.; 2004 253 p.

[42] Dagorn L, Petit M, Stretta JM. Simulation of large-scale tropical tuna movements in relation with daily remote sensing data: the artificial life approach. Bio Systems. 1997;44(3):167–80.; 9460558. 10.1016/s0303-2647(97)00051-8 9460558

[43] Belkin IM, O'Reilly JE. An algorithm for oceanic front detection in chlorophyll and SST satellite imagery. J Mar Systems. 2009;78(3):319–26. 10.1016/j.jmarsys.2008.11.018

[44] Prince ED, Ortiz M, Venizelos A, Rosenthal DS. In-water conventional tagging techniques developed by the cooperative tagging center for large, highly migratory species. American Fisheries Society Symposium. 2002;30:155–71.

[45] Kraus RT, Rooker JR. Patterns of vertical habitat use by Atlantic blue marlin (*Makaira nigricans*) in the Gulf of Mexico. Gulf Car Res. 2007;19(2):89–97.

[46] Hoolihan JP, Luo J, Goodyear CP, Orbesen ES, Prince ED. Vertical habitat use of sailfish (*Istiophorus platypterus*) in the Atlantic and eastern Pacific, derived from pop-up satellite archival tag data. Fish Oceanogr. 2011;20(3):192–205. 10.1111/j.1365-2419.2011.00577.x

[47] Teo SLH, Boustany A, Blackwell S, Walli A, Weng KC, Block BA. Validation of geolocation estimates based on light level and sea surface temperature from electronic tags. Mar Ecol Prog Ser. 2004;283:81–98.; 6146205.

[48] Domeier ML, Kiefer D, Nasby-Lucas N, Wagschal A, O'Brien F. Tracking Pacific bluefin tuna (*Thunnus thynnus orientalis*) in the northeastern Pacific with an automated algorithm that estimates latitude by matching sea-surface-temperature data from satellites with temperature data from tags on fish. Fish Bull. 2005;103:292–306.

[49] Farneti R, Delworth TL, Rosati AJ, Griffies SM, Zeng F. The Role of Mesoscale Eddies in the Rectification of the Southern Ocean Response to Climate Change. J Phys Oceanogr. 2010;40(7):1539–57. 10.1175/2010JPO4353.1; 13703188.

[50] Hallberg R. Using a resolution function to regulate parameterizations of oceanic mesoscale eddy effects. Ocean Modelling. 2013;72:92–103.

[51] O'Hern JE, Biggs DC. Sperm Whale (Physeter macrocephalus) Habitat in the Gulf of Mexico: Satellite Observed Ocean Color and Altimetry Applied to Small-Scale Variability in Distribution. Aquatic Mammals. 2009;35(3):358–66.

[52] Michaels AF. Highly active Eddies. Science. 2007;316(5827):992–3. 17510353 10.1126/science.1140059

[53] Worm B, Lotze HK, Myers RA. Predator diversity hotspots in the blue ocean. Proc Natl Acad Sci USA. 2003;100:9884–8. 12907699 10.1073/pnas.1333941100 PMC187874

[54] Rooker JR, Simms JR, Wells RJD, Holt SA, Holt GJ, Graves JE, et al Distribution and habitat associations of billfish and swordfish larvae across mesoscale features in the Gulf of Mexico. PloS ONE. 2012;7(4):e34180; 22509277. 10.1371/journal.pone.0034180 22509277 PMC3324529

[55] Maul GA, Williams F, Roffer MA, Sousa FM. Remotely sensed oceanographie patterns and variability of bluefin tuna catch in the Gulf of Mexico. Oceanologica ACTA. 1984;7:469–79.

[56] Bakun A. Fronts and eddies as key structures in the habitat of marine fish larvae: opportunity, adaptive response and competitive advantage. Scientia Marina (Barcelona). 2006;70:105–22.; 7151124.

[57] Solanki HU, Mankodi PC, Nayak, Somvanshi VS. Evaluation of remote-sensing-based potential fishing zones (PFZs) forecast methodology. Continental shelf research. 2005;25(18):2163–73.; 7791194.

[58] Druon JN, Fromentin JM, Aulanier F, Heikkomen J. Potential feeding and spawning habitats of Atlantic bluefin tuna in the Mediterranean Sea. Mar Ecol Prog Ser. 2011;439:223–40.

[59] Travis J, Coleman FC, Auster PJ, Cury PM, Estes JA, Orensanz J, et al Integrating the invisible fabric of nature into fisheries management (Proceedings of the National Academy of Sciences of the United States of America (2014) 111, 2 (581–584) 10.1073/pnas.1305853111). Proc Natl Acad Sci USA. 2014;111(12):4644. 10.1073/pnas.1305853111 PMC3896161 24367087

[60] Twatwa NM, van der Lingen CD, Drapeau L, Moloney CL, Field JG. Characterising and comparing the spawning habitats of anchovy *Engraulis encrasicolus* and sardine *Sardinops sagax* in the southern Benguela upwelling ecosystem. Afr J Mar Sci. 2005;27(2):487–99.

[61] Muhling BA, Lamkin JT, Roffer MA. Predicting the effects of climate change on bluefin tuna *(Thunnus thynnus*) spawning habitat in the Gulf of Mexico. ICES J Mar Sci. 2011;68:1051–62.

